# Multi-Mode Ultrasonic Guided Waves Based Damage Detection in L-Bars with Asymmetric Cross-Section with Sum of Multiple Signals Method

**DOI:** 10.3390/s22030922

**Published:** 2022-01-25

**Authors:** Zhengyan Yang, Jiaqi Zhang, Kehai Liu, Yuebin Zheng, Shuyi Ma, Zhanjun Wu

**Affiliations:** 1 College of Transportation Engineering, Dalian Maritime University, Dalian 116024, China; zyyang1993@dlmu.edu.cn; 2 State Key Laboratory of Structural Analysis for Industrial Equipment, Dalian University of Technology, Dalian 116024, China; wuzhj@dlut.edu.cn; 3 Songshan Lake Materials Laboratory, Institute of Physics, Chinese Academy of Sciences, Dongguan 523808, China; liukehai@sslab.org.cn (K.L.); zhengyuebin@sslab.org.cn (Y.Z.); 4 School of Traffic and Transkportation, Dalian University of Science and Technology, Dalian 116052, China; dlut_msy@163.com

**Keywords:** damage detection, ultrasonic guided wave, multi-mode characteristic, sum of multiple signals, excitation optimization, weighted gathering

## Abstract

Bars are significant load-carrying components in engineering structures. In particular, L-bars are typical structural components commonly used in truss structures and have typical irregular asymmetric cross-sections. To ensure the safety of load-carrying bars, much research has been done for non-destructive testing (NDT). Ultrasonic guided waves have been widely applied in various NDT techniques for bars as a result of the long-range propagation, low attenuation, and high sensitivity to damages. Though good for inspection of ultrasonic guided waves in symmetric cross-section bar-like structures, the application in asymmetric ones lacks further research. Moreover, traditional damage detection in bars using ultrasonic guided waves usually depends on a single-mode at a lower frequency with lower sensitivity and accuracy. To make full use of all frequencies and modes, a multi-mode characteristic-based damage detection method is presented with the sum of multiple signals (SoM) strategy for L-bars with asymmetric cross-section. To control the desired mode in multi-mode ultrasonic guided waves, excitation optimization and weighted gathering are carried out by the analysis of the semi-analytical finite element (SAFE) method and the normal mode expansion (NME) method. An L-bar example with the asymmetric cross-section of 35 mm × 20 mm × 3 mm is used to specialize the proposed method, and some finite element (FE) models have been simulated to validate the mode control. In addition, one PZT is applied as a contrast in order to validate the multielement mode control. Then, more FE simulations experiments for damage detection have been performed to validate the damage detection method and verify the improvement in detection accuracy and damage sensitivity.

## 1. Introduction

Load-carrying structures are facing challenges from a complex physical environment during service. To assure the integrity of in-service engineering facilities, much work has been done for the inspection of complex load-carrying structures. The occurrence of structural damage is able to threaten the safety of structures and further lead to unacceptable catastrophic consequences, calling for the development of non-destructive testing (NDT) [1] and structural health monitoring (SHM) [2]. The current state of diverse methods for NDT and SHM has been reviewed carefully in [3], among various methods ultrasonic guided wave technology has been widely utilized because of the excellent stability over long propagation and high sensitivity to damage initiation [4,5].

Ultrasonic guided waves have been applied for the rapid long-range damage detection in common structures such as plates [6], pipes [7] and composite [8]. And among various embedded sensors for the above structures, piezoelectric transducers (PZT) are popularly used as stable sensors thanks to their piezoelectric effect for surface tractions [9]. Giurgiutiu et al. [10] described a model of the ultrasonic guided waves generation and detection mechanism with PZTs in plate-like structures, which was utilized to design excitation for mode selection. Raghavan et al. [2,11] modeled transient ultrasonic guided wavefields excited by differently shaped PZTs and developed the rigorous analytical solutions based on 3D linear elasticity, which was utilized for the optimum dimensional design of transducers. Considering the strong material damping and anisotropic characteristic, Mei et al. [12] investigated the ultrasonic guided wave excitation and propagation by semi-analytical finite element (SAFE) method, and the theoretical predictions were experimentally validated using PZT excitation. By employing the normal mode expansion (NME) method, Ditri et al. [13] solved the generation of axisymmetric ultrasonic guided wave modes in hollow cylinders by surface loading, and the mode amplitude factors were obtained. Li et al. [14] extended Ditri’s work for the NME method and studied the damage detection of both distance and circumferential location in a hollow cylinder. The theoretical approaches are convenient and efficient in simple geometries, however, the excitation and sensing modal of an ultrasonic guided wave for damage detection is more complicated in complex structures.

Arbitrary cross-section bars are key elements in engineering structures as major load-carrying structures. During the past two decades, research of damage detection for bars with complex arbitrary cross-sections such as rails via ultrasonic guided waves has attracted a lot of attention. Zhou et al. [15] developed a ToF based 3D diagnostic imaging method using the active sensor network, capable of real-time inspecting complex rails, and the ToF-based features were extracted from acquired signals to develop field values of source images. Owing to the complex multi-mode characteristic of arbitrary cross-section structures, the wave propagation becomes particularly intricate [16]. The SAFE method can be employed to understand the multi-mode characteristic by modeling the acoustoelastic theory in arbitrary cross-section bars [17]. Based on the propagation characteristic analysis by SAFE method, Li et al. [18] proposed a probability-based diagnostic image method to identify defect locations in H-bars with symmetric excitation; Yu et al. [19] presented a rapid screening method for the bond line between a T-bar and composite panel using feature guided waves; our research team also studied the damage localization and stress monitoring of T-bars in previous work [20,21]. However, these studies mostly focus on bars with irregular symmetric cross-sections, and the multi-mode characteristic in bars with asymmetric cross-sections will be more complex because of the more diverse mode wave structures. In particular, L-bars with irregular asymmetric cross-sections are commonly used in engineering structures. Therefore, in this study, we research damage detection in L-bars with an asymmetric cross-section of 35 mm × 20 mm × 3 mm using ultrasonic guided waves.

In addition, only the single mode of ultrasonic guided waves at lower frequency is used to identify the axial damage location because of the mode wave structure vibrating on the whole cross-section. However, low-frequency detection can be conditioned by low sensitivity and accuracy due to longer wavelength and wider signal bandwidth, respectively [22]. To make full use of all frequencies and modes, a well-established sum of multiple signals (SoM) method is widely applied to phased array beamforming [23], diagnostic imaging [24], delay and sum [25]. The purpose of our work is to improve the sensitivity and accuracy of damage detection using multi-mode ultrasonic guided waves in L-bars with asymmetric cross-section, thus the SoM method coupled with mode control is proposed. The selection of frequencies and modes, optimization of wave exciting, and weighted gathering of signal receiving is applied to realize mode control on the basis of SAFE and NME. In this paper, we first expand on the process of damage detection in Section 2. Then, the methodology is specialized to an L-bar example following the process in a logical and detailed manner. Finally, we further carry out experimental tests to evaluate the effectiveness of the proposed method.

## 2. Methodology

**Figure 1:**
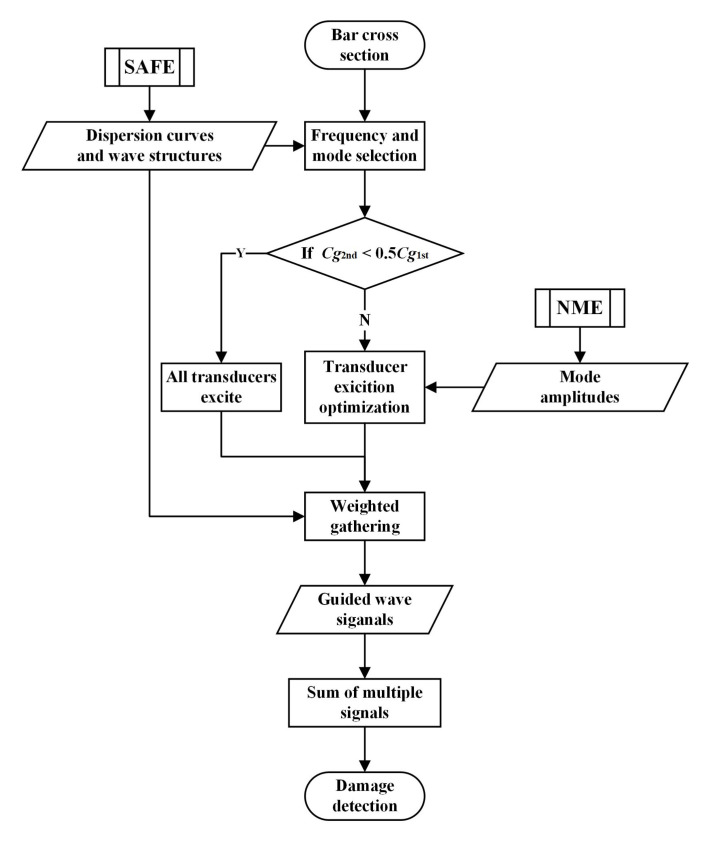
Process of the proposed method.

The defect detection method developed in this paper is primarily oriented toward L-bars with asymmetric cross-section, relying on the sum of multiple ultrasonic guided wave signals. And the specific procedure of the methodology is illustrated in Figure 1.

Firstly, frequencies and modes are selected for damage detection of L-bars. The semi-analytical finite element (SAFE) method is used to investigate the multi-mode characteristic of ultrasonic guided waves in L-bars with asymmetric cross-section, allowing for dispersion curves and wave structures. Then, appropriate frequencies can be selected because they are approximate nondispersive seeing the flat group velocity dispersion curves. The fastest modes at given frequencies are later chosen correspondingly.

Secondly, a strategy for mode control is established in view of the excitation and acquisition of ultrasonic guided waves. The excitation optimization could be applied if the group velocities of the fastest mode are not more than twice as fast as any mode else, which criterion can be expressed by the relation $Cg_{2\mathrm{nd}}>0.5Cg_{1\mathrm{st}}$. The combination of excitation transducers can be optimized relying on the mode amplitudes obtained by normal mode expansion (NME) analysis. Then for purer ultrasonic guided wave signal with the desired mode, a weighted gathering method is used to superpose weighted outputs of receiving transducers, where the weight of each transducer is calculated according to wave structures.

Finally, the propagation time is transferred into the distance on the basis of the propagation velocity acquired from dispersion curves. Then, all ultrasonic guided wave signals at selected frequencies with different modes are summed to form the damage signal. The damage diagnosis is carried out by relying on the damage index (DI), the ratio of damage echo amplitude to excitation peak amplitude in the damage signal Hilbert envelope. Sequentially the damage location is pointed out by the time of flight (ToF).

## 3. Validation with L-Bar Example

The main aim of the following work is to validate the proposed method with SoM of multi-mode ultrasonic guided waves in L-bars, thus we specialize the methodology to an L-bar example with a cross-section of 35 mm × 20 mm × 3 mm in this section. On the basis of this specification, other L-bars with different dimensions could be analyzed and detected according to the following, despite the variation of frequency, mode, excitation, and reception.

### 3.1. Selection of Frequencies and Modes of Ultrasonic Guided Waves

A few sample problems have been argued by Rose to exhibit the significance of frequency and mode selection in ultrasonic detection [26]. Thus we accomplish the selection of frequencies and modes for the damage detection of an L-bar based on the multi-mode characteristic of ultrasonic guided waves. To investigate the multi-mode characteristic, dispersion curves and wave structures of an L-bar example can be analytically obtained by the SAFE method [27,28].

**Figure 2:**
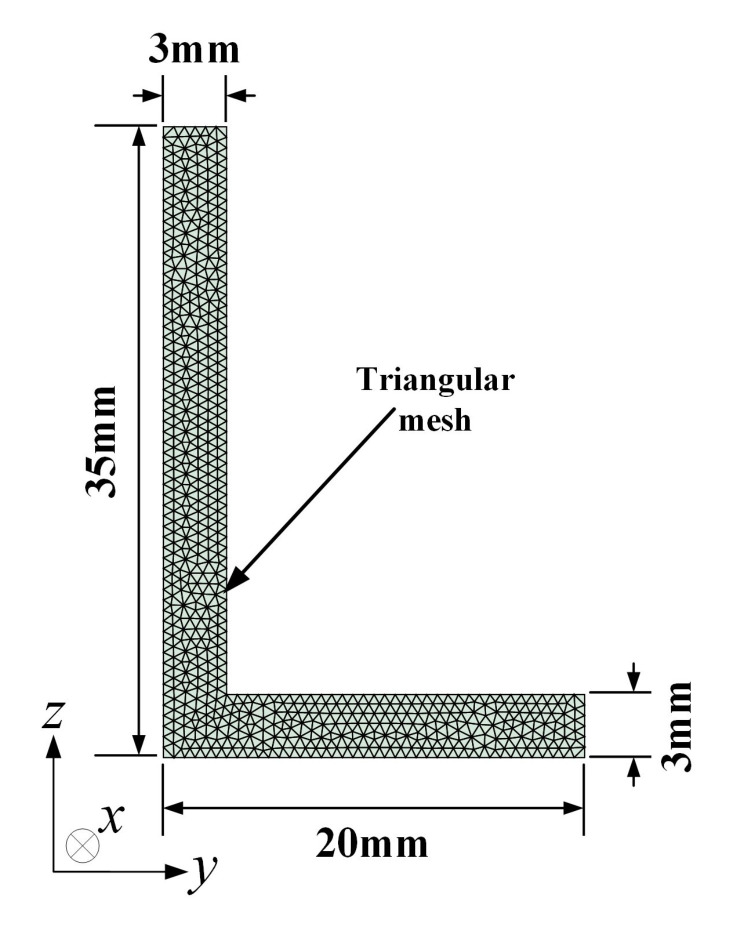
Cross-section diagram of the SAFE model for a L-bar.

Considering the SAFE method applied to structures with arbitrary cross-section has been derived detailedly in [29], only the pivotal processes used in this work are next explained in brief. Figure 2 displays the two-dimension schematic of an aluminum L-bar cross-section which is meshed by 1198 triangular elements with the size of 0.5 mm. The mass density is 2700 kg/m${}^{3}$, Young’s modulus is 71 Gpa, and Poisson’s ratio is 0.33.

When the vibration field is assumed harmonic along the propagation direction, the SAFE eigenvalue equation is disposed of as

(1) $$\left[K_{1}+ikK_{2}+k^{2}K_{3}-\omega^{2}M\right]\tilde{U}=0,$$

where *i* is imaginary unit, $\tilde{U}$ is eigenvector, $K_{1}$, $K_{2}$, $K_{3}$ are stiffness matrices, M is mass matrix. These regular matrices can be calculated by the material properties and geometry [29]. The two variables *k* and ω=2πf are wave number and circular frequency, respectively. Equation (1) can be solved by firstly fixing the wave number *k* and then solving for the frequency ω, that is, the frequencies $f_{n}$ can be calculated for required wavenumber values $k_{n}$ [30]. Sequentially, the piecewise cubic Hermite interpolant [31] is employed to capture the wavenumber approximations at required frequency values. Finally, the group velocities are calculated in the light of the formula [32]

(2) $$Cg=\frac{d\omega }{dk}.$$

The SAFE method is implemented by the code in MATLAB© platform, and the numerical procedure is schematized in Appendix A.1.

**Figure 3:**
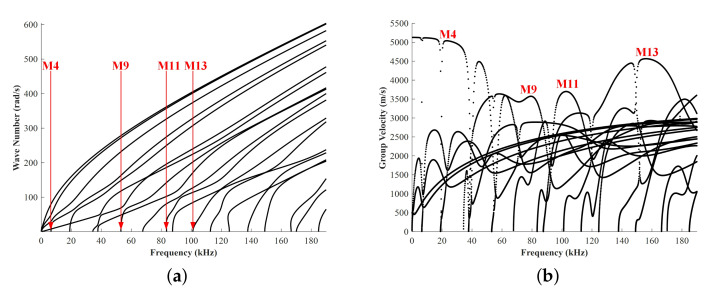
Dispersion curves for waves propagating in a L-bar: (**a**) wave number, (**b**) group velocity.

Figure 3 shows the dispersion curves for ultrasonic guided waves in the L-bar. Similar to ultrasonic guided waves in rails [33], the modes can be numbered as M1, M2, M3, etc. in order of the frequency at starting points. It is observed that at frequencies of 25, 80, 100, and 155 kHz, the fastest mode is approximate nondispersive because of the flat group velocity dispersion curve [34]. The fastest modes are correspondingly M4, M9, M11, and M13, respectively.

### 3.2. Mode Control

**Figure 4:**
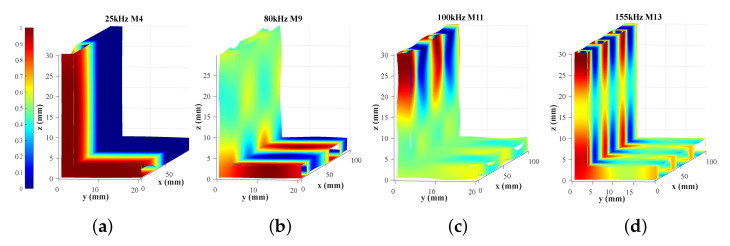
Normalized mode displacement shapes in the longitudinal *x* calculated by the SAFE method: (**a**) 25 kHz M4, (**b**) 80 kHz M9, (**c**) 100 kHz M11, (**d**) 155 kHz M13.

After the aforementioned analysis of the propagation characteristics in L-bars, we have selected the approximate frequencies and modes for SoM of multi-mode ultrasonic guided waves. However, the signal of the desired mode is subject to other modes due to the multi-mode characteristic. Therefore, to avoid the effect of other modes rather than the only desired one in received signals, the mode control should be performed on the account of wave structures. Wave structures can be analytically procured according to dispersion curves and SAFE model [28], and those of the L-bar can be characterized by normalized mode shapes as shown in Figure 4. The vibration displacement in Figure 4 is the in the longitudinal *x*, the direction of propagation because the longitudinal vibration is vulnerable to damages in the cross-section [20]. Based on the wave structures information, methods conduced to mode control will be proposed in view of the excitation and reception of ultrasonic guided waves, respectively, in the following paragraphs.

The combination of exciting transducers can be optimized by the NME method so as to excite the desired mode as the major mode in ultrasonic guided wave signals. Because the deduction of the NME method for excitation in arbitrary cross-section structures has been detailed in [35], only the equations used in this section are introduced here. We consider the complex reciprocity relation [36],

(3) $$\nabla \cdot \;\left(v_{n}^{*}\cdot T_{m}+v_{m}\cdot T_{n}^{*}\right)\;=0,$$

where $T_{m}$, $v_{m}$ and $T_{n}$, $v_{n}$ are the stress field and particle velocity governing two different solutions of the propagation of ultrasonic guided waves, besides, the asterisk ${}^{*}$ represents complex conjugation. Then, invoking traction free boundary conditions and the Gauss divergence theorem, the orthogonal condition can be obtained

(4) $$\int_{s}\left(v_{n}^{*}\cdot T_{m}+v_{m}\cdot T_{n}^{*}\right)ds=0,$$

where *s* is the cross-section.

With the above orthogonal condition, we can find the amplitudes of each mode resulting from prescribed loadings on the boundary of the cross-section. Firstly, the actual fields are given as

(5) $$T_{m}=\underset{m}{\sum }A_{m}\left(x\right)T_{m}\left(y,z\right),v_{m}=\underset{m}{\sum }A_{m}\left(x\right)v_{m}\left(y,z\right).$$

Considering axial loadings $p_{1}\left(x\right)$ and outward normal loadings $p_{2}\left(y,z\right)$ on the boundary of the L-bar, i.e.,

(6) $$T\cdot n=-p_{1}\left(x\right)p_{2}\left(y,z\right)\gamma ,\left(\gamma =x,n\right),$$

the mode amplitude of mode *m* can be expressed through a series of derivations [35]

(7) $$A_{m}\left(x\right)\;=-\frac{e^{-jk_{m}x}}{4P_{m}}\langle p_{1}\left(x\right),e^{jk_{m}x}\rangle \langle p_{2}\left(y,z\right),v_{m\gamma }^{*}\rangle ,$$

where $P_{m}$ is the power flow of mode *m*

(8) $$P_{m}=-\frac{1}{4}\int_{s}{\left(v_{m}^{*}\cdot T_{m}+v_{m}\cdot T_{m}^{*}\right)}_{x}ds,$$

$\langle p_{1}\left(x\right),e^{jk_{m}x}\rangle$ and $\langle p_{2}\left(y,z\right),v_{m\gamma }^{*}\rangle$ are the axial and cross-section amplitude factors, respectively.

(9) $$\begin{array}{cc} \langle p_{1}\left(x\right),e^{jk_{m}x}\rangle & =\int_{-L}^{L}e^{jk_{m}x}p_{1}\left(x\right)dx,\\ \langle p_{2}\left(y,z\right),v_{m\gamma }^{*}\rangle & =\oint_{l}p_{2}\left(y,z\right)v_{m}^{*}\left(y,z\right)\cdot \gamma dl. \end{array}$$

Here surface loadings are given as

(10) $$p_{1}\left(x\right)\;=P_{1},\;\left|x\right|\;\leq L_{t},\;p_{2}\left(y,z\right)\;=P_{2},\left(y,z\right)\;\in l.$$

Substituting Equation (10) into Equation (9), the the axial and cross-section amplitude factors can be calculated

(11) $$\langle p_{1},e^{jk_{m}x}\rangle =\frac{2P_{1}\sin\left(k_{m}L_{t}\right)}{k_{m}}$$

(12) $$\langle p_{2}\left(y,z\right),v_{m\gamma }^{*}\rangle =\left\{\begin{array}{c} P_{2}\oint_{l}v_{mx}^{*}\left(y,z\right)dl,\;\gamma =x\\ P_{2}\oint_{l}v_{m}^{*}\left(y,z\right)\;\cdot \;ndl,\;\gamma =n \end{array}\right.$$

where

(13) $$v_{m}^{*}\left(y,z\right)\;\cdot \;n=v_{my}^{*}n_{y}+v_{mz}^{*}n_{z}$$

**Figure 5:**
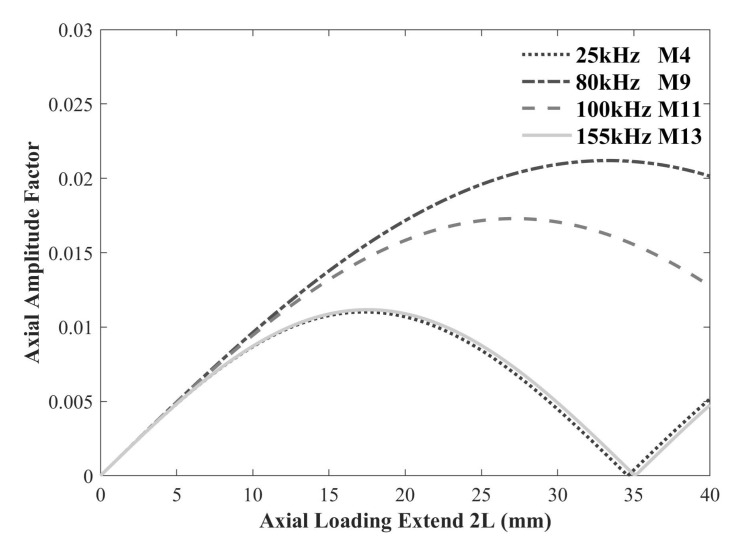
Axial amplitude factor varying with extending $2L_{t}$.

According to Equation (11), the axial amplitude factor is a function of wavenumber $k_{m}$ and loading length $L_{t}$, which is shown in Figure 5. For a selected frequency $k_{m}$ is fixed, therefore, $L_{t}$ is the optimum loading length when the axial amplitude factor reaches the maximum. We take $2L_{t}=18$ mm as the loading length for the following studies on account of its suitability for all four modes.

**Figure 6:**
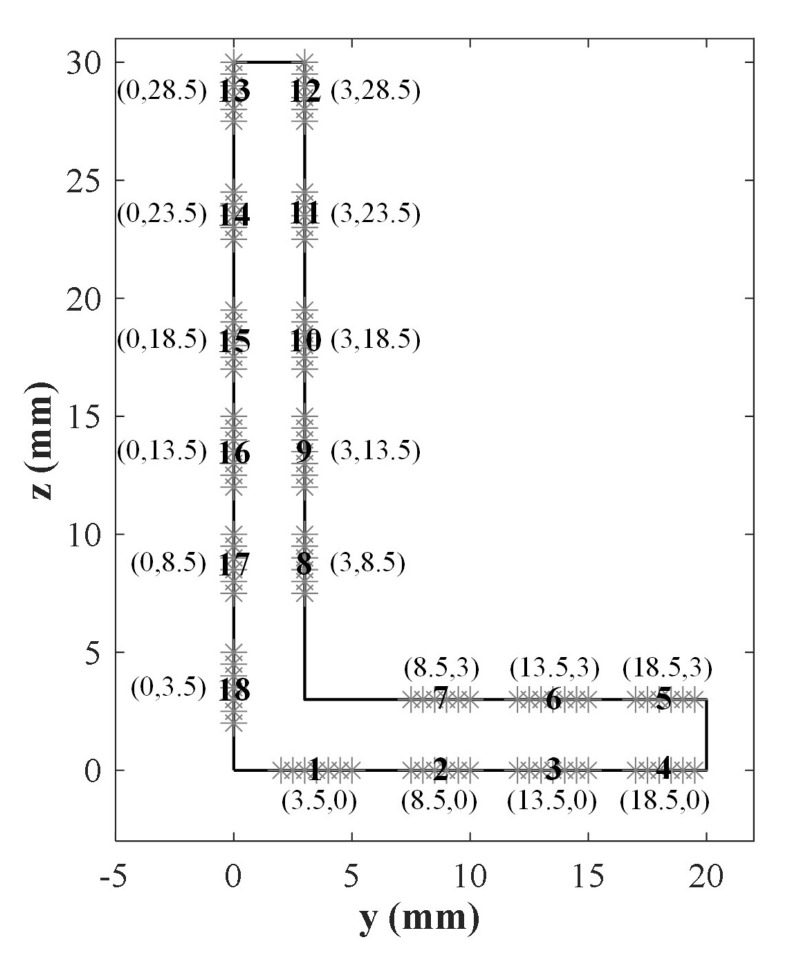
Multielement loading layout for the L-bar, and all loading regions are numbered.

Such as the research in [37], partial loadings are applied to the edge of the cross-section composed of multielement segments rather than the whole boundary. Figure 6 shows a set of loadings regarded as transducers with a width of 3 mm, and all 18 loading elements are numbered. The mode amplitudes of any combination of all loading elements can calculate to screen out the optimal excitation combination.

**Figure 7:**
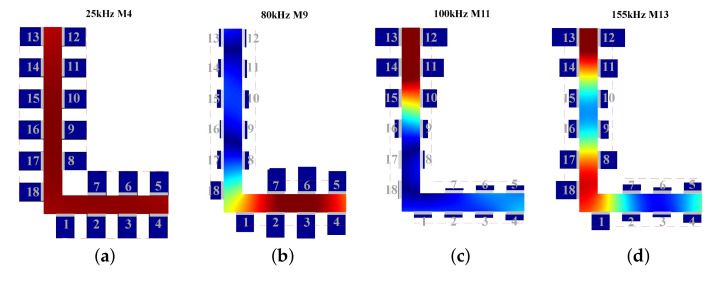
Normalized weight of each transducer: (**a**) 25 kHz M4, (**b**) 80 kHz M9, (**c**) 100 kHz M11, (**d**) 155 kHz M13.

Combining with mode shapes in Figure 4, the displacements in the transducer position can be integrated as the weight for *i*th transducer

(14) $$w_{i}=\overset{N}{\underset{j=1}{\sum }}u_{j},$$

where $u_{j}$ is the displacement of *j*th node in *i*th transducer position, and *N* is the number of nodes. We calculate the weights of 25 kHz M4, 80 kHz M9, 100 kHz M11, and 155 kHz M13 in sequence, and normalize these weights severally. Figure 7 shows normalized weights of all transducers at different frequencies, the height of the histogram represents the value of normalized weight. And the mean value can be calculated (red dotted line).

**Figure 8:**
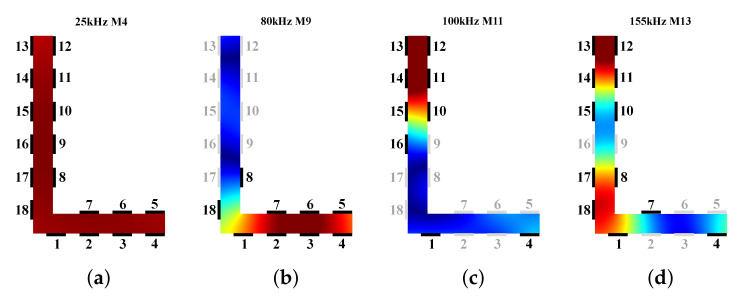
Optimized excitation combinations (black ones): (**a**) 25 kHz M4, (**b**) 80 kHz M9, (**c**) 100 kHz M11, (**d**) 155 kHz M13.

**Figure 9:**
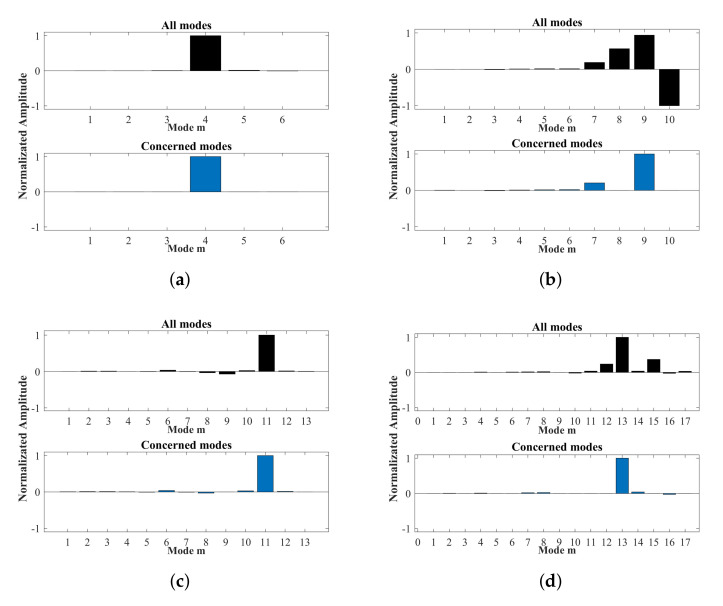
Normalizated amplitudes of modes: (**a**) 25 kHz M4, (**b**) 80 kHz M9, (**c**) 100 kHz M11, (**d**) 155 kHz M13.

Then substituting $2L_{t}=18$ mm, $P_{1}=P_{2}=1$ and all multielement loading combinations from the distribution in Figure 6, based on the Equation (7), Equations (11) and (12), the axial mode amplitudes of all modes are calculated. The calculation is implemented by MATLAB© code that is introduced briefly in Appendix A.1. Sequentially, the optimal combination of transducers can be selected according to the criterion of max amplitude ratio between the desired mode to secondary mode. Here we eliminate the modes in which group velocities are lesser than the half of group velocity of the desired mode due to their little effect on the desired mode applied to damage detection. In order to ensure the excitation energy, the transducers with the weight above the mean value are picked out from Figure 7 for excitation optimization. The optimal combinations for the selected ultrasonic guided waves of the last section are screened out as displayed in Figure 8, which normalized amplitudes are shown in Figure 9.

Note that the condition 25 kHz M4 does not need excitation optimization thanks to the group velocity of M4 being more than twice as fast as any mode else. On the contrary, optimal combinations of transducers need to be screened out in the other conditions 80 kHz M9, 100 kHz M11, and 155 kHz M13.

The weighted gathering method can be used for signal receiving to obtain cleaner signals. To extract the desired prime modes, the signals received by transducers are superposed after weighting each signal with the corresponding stacking weight. The gathering signal can be expressed by

(15) $$sg=\overset{n}{\underset{i=1}{\sum }}s_{i}w_{i},$$

where *n* is the number of the transducers, $s_{i}$ is the signal received by *i*th transducer and $w_{i}$ is the weight calculated by mode shapes and transducer positions. For the transducer layout in Figure 6, Table 1 lists the normalized weights of all transducers at different frequencies.

### 3.3. FE Simulation

**Figure 10:**
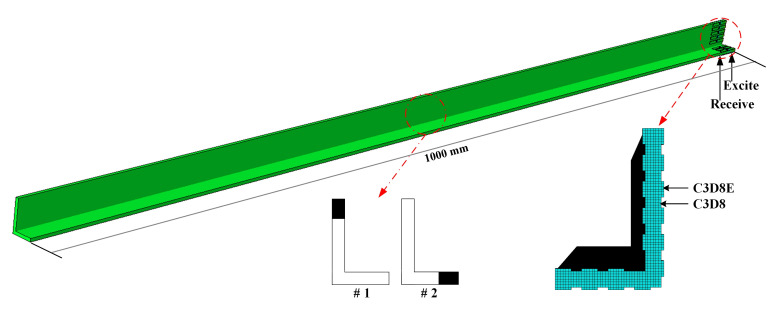
The FE model consisting of an L-bar and PZTs.

To demonstrate the effectiveness of the above mode control for the proposed method, a finite element (FE) model of the L-bar is established by a commercial FE software, ABAQUS©, as shown in Figure 10 and Table 2. The length of this L-bar model is 1000 mm, moreover, the sectional dimension and material properties are the same as the preceding SAFE model. Thanks to the wide application of PZT as actuators and sensors of ultrasonic guided waves in structural health monitoring [9], simulated PZTs (18 mm × 3 mm × 0.5 mm) are mounted on the surface of the bar in this work. The material properties of PZTs are shown in Table 3. The excitation and acquisition are based on piezoelectric property: medium produces electricity when under mechanical stress, called the piezoelectric effect; conversely, applying excitation electric field, medium generates mechanical deformation, called the inverse piezoelectric effect [38].

**Figure 11:**
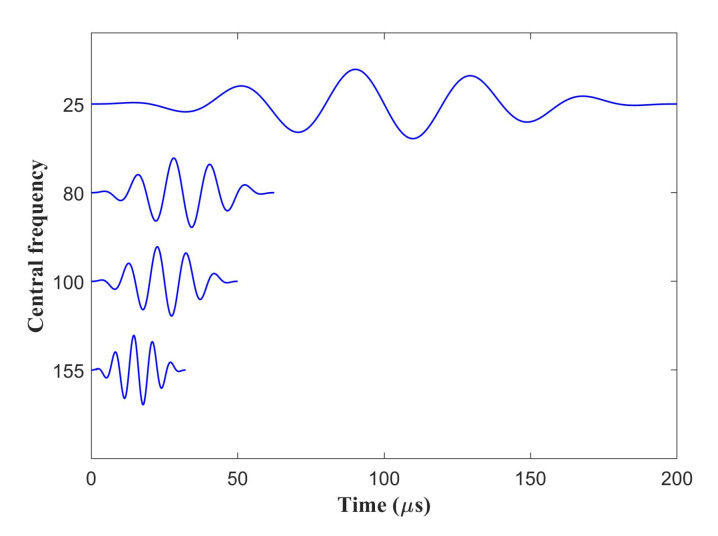
The Hanning windowed sinusoidal signals.

The model element sizes of PZT and L-bar are 0.5 mm and 2 mm, respectively, which followed the criterion of arranging 10–20 elements per wavelength [39]. The 8-node linear brick elements (C3D8) and 8-node linear piezoelectric brick elements (C3D8E) applied to our FE simulation are provided in ABAQUS [40]. The elements are C3D8 for the L-bar, and C3D8E for PZTs in order to achieve the piezoelectric effect [20,21]. The exciting PZTs for these four frequencies are the designed combinations in the last section as shown in Figure 8. 5-cycle Hanning windowed sinusoidal signals with central frequencies of 25, 80, 100, and 155 kHz are respectively inputted as excitation signals, as shown in Figure 11, and the amplitude is normalized. The Hanning window modulation function is

(16) $$w\left(n\right)=\frac{1}{2}\left[1-\cos\left(\frac{2\pi (n-1)}{N}\right)\right],0\leq n\leq N.$$

**Figure 12:**
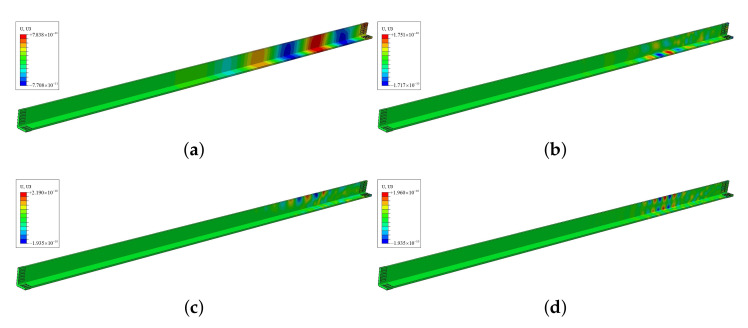
Static images during the propagation of guided waves in FE at: (**a**) 25 kHz M4, (**b**) 80 kHz M9, (**c**) 100 kHz M11, (**d**) 155 kHz M13.

**Figure 13:**
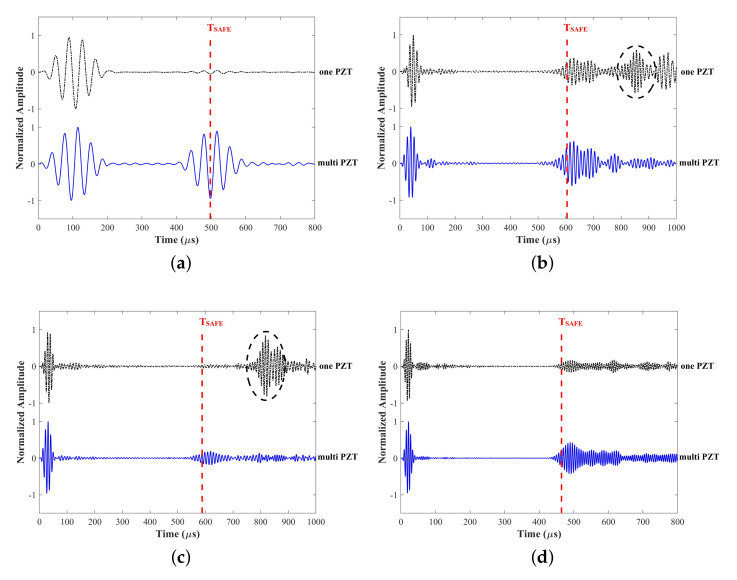
Time-domain ultrasonic guided wave signals in FE via only one PZT and weighted gathering at: (**a**) 25 kHz M4, (**b**) 80 kHz M9, (**c**) 100 kHz M11, (**d**) 155 kHz M13.

**Figure A1:**
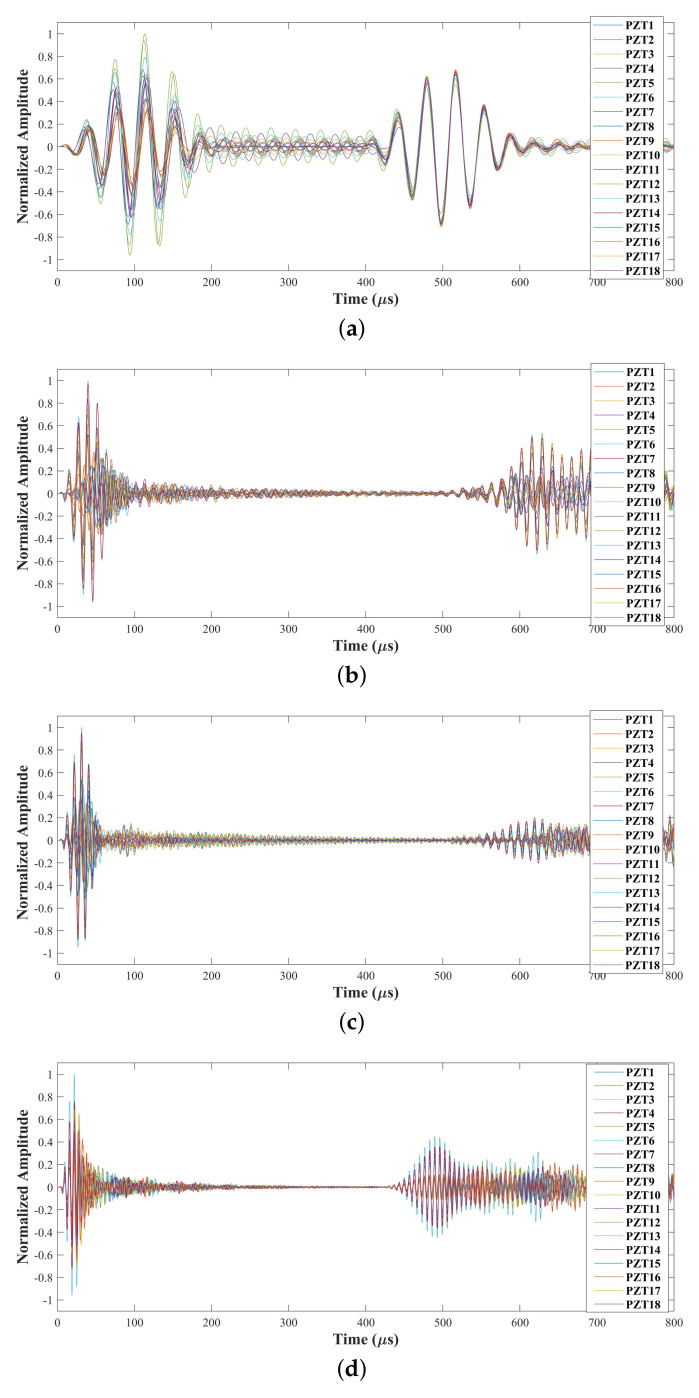
FE outputs of all PZTs at: (**a**) 25 kHz M4, (**b**) 80 kHz M9, (**c**) 100 kHz M11, (**d**) 155 kHz M13.

The static displacement nephograms of all excitations are shown in Figure 12, where the mode shapes are much the same as wave structures. The displacements of 25 kHz are distributed throughout the whole “L” section. Furthermore, the displacements of 80 kHz are concentrated in the short part of “L”, conversely, the displacements of 100 and 155 kHz are concentrated in the long part of “L”. Figure A1 in Appendix A.2 shows outputs of every receiving PZT, then signals of ultrasonic guided waves can be acquired by the weighted superposing the outputs of PZTs, as shown in Figure 13.

In addition, to validate that the multielement excitation optimization and weighted gathering above are reasonable and effective, we choose only one PZT which weight is shown in Table 1 to excite and receive ultrasonic guided waves as a contrast. PZT-16, PZT-6, PZT-12, and PZT-13 are for four different, respectively. The propagation time calculated by group velocity from SAFE is shown as the red dotted line in Figure 13. It is observed that the wave energy is lower and not enough for damage detection at some frequencies as Figure 13a,b; the proportion of the desired mode is too low to be applied as Figure 13b,c. On the contrary, the results indicate that the multi PZTs applied for excitation optimization and weighted gathering can avoid low energy and proportion of the desired mode.

Some notches are set as damages in the model individually with the length of 10 mm and the width of 2 mm as #1, #2 in Figure 10, which are respectively in the long and short parts of the “L” section. The location is the middle of the bar, where the longitudinal distance between notch and excitation is 500 mm. And the displacements on the bar surface are received by PZTs.

### 3.4. Sum of Multiple Signals

After the mode control based on the optimization design of excitation and reception, the damage signal can be obtained by summed ultrasonic guided wave signals at all frequencies. The propagation distance for each frequency can be calculated by the propagation velocity.

(17) $$2L=Cg\left(t-\frac{T}{2}\right),$$

where Cg is the propagation velocity interpolated from the group velocity dispersion curve, *T* is the time duration of the exciting signal.

To avoid the elimination of the phase in SoM, we sum the Hilbert envelopes instead of the signals to constitute the damage signal. The Hilbert transform of the signal $f\left(t\right)$ is defined as

(18) $$H\left(t\right)\;=\frac{1}{\pi }\int_{-\infty }^{+\infty }\frac{f\left(t^{\prime }\right)}{t-t^{\prime }}dt^{\prime }$$

**Figure 14:**
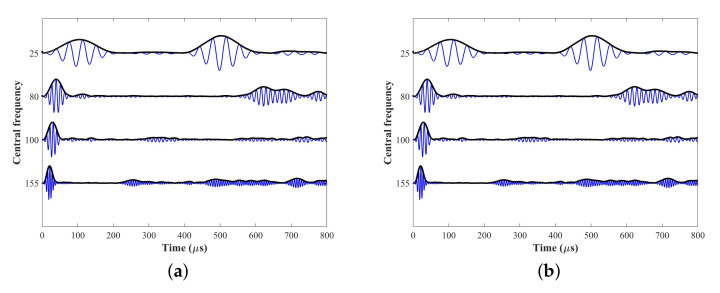
Time-domain signals from damaged FE model at all four frequencies: (**a**) #1, (**b**) #2.

**Figure 15:**
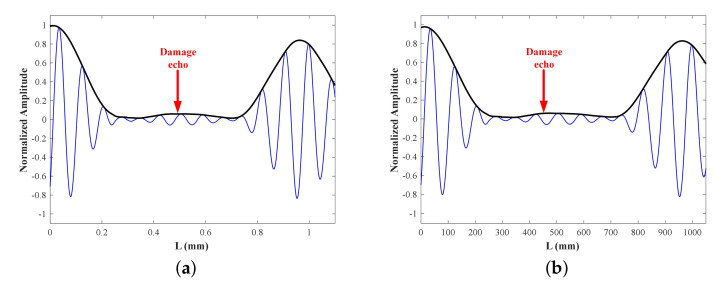
Damage signals from damaged FE model only at 25 kHz: (**a**) #1, (**b**) #2.

**Figure 16:**
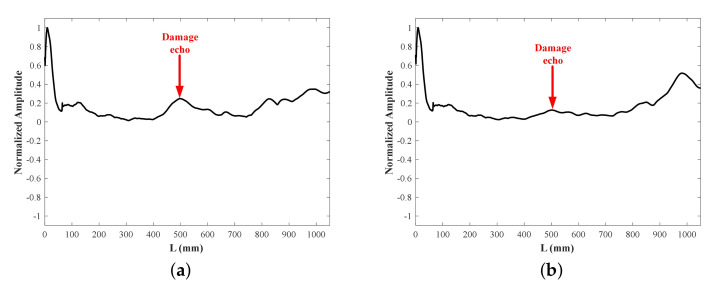
SoM damage signals from damaged FE model: (**a**) #1, (**b**) #2.

Referring to pipes [41], the damage location is pointed out by the time of flight (ToF). ToF is determined as the time period between the times of excitation peak and damage echo extracted from the Hilbert envelope of the damage signal. Figure 14 shows time-domain signals at all four frequencies under two cases of the damaged L-bar FE model, i.e., damage #1 and damage #2, as stated in Figure 10. Figure 15 and Figure 16 show damage signals of 25 kHz and SoM respectively, where time is converted to distance. Damage can be located by finding out the apex of the damage echo wave packet. The damage locations are listed in Table 4 where we can obviously figure out that the SoM can effectively improve the detection accuracy.

## 4. Experiments

### 4.1. Experimental Setup

**Figure 17:**
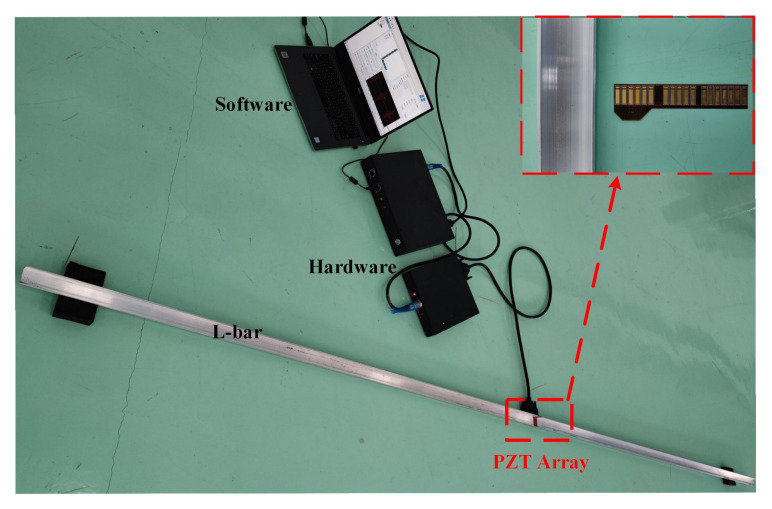
The experimental setup.

Figure 17 shows the experimental setup for damage detection in an aluminum L-bar with the length of 2000 mm and the same cross-section as Figure 2. Thirty-six PZTs are glued on the surface in the middle of the L-bar resulting in two circle arrays as the exciting and receiving transducer arrays respectively. These PZTs (P51,d31) are supported by Wuxi Huifeng Electronic Co, Ltd. (Wuxi, China), the length is $2L_{t}=12$ mm that basically meets the requirements of excitation according to Figure 5. The layout and size of PZTs are set the same as Figure 10.

A system named Guided-wave Diagnostic Platform V1.0 supported by Dalian SunRising Technologies Ltd. (Dalian, China) is used to perform the excitation and acquisition process for ultrasonic guided waves. The system is composed of software including ARM master controller, FPGA slave controller; and hardware integrated with central control unit, signal generator, power amplifier, 64-channel change-over switch, a data acquisition unit, etc. The sampling rate is 12 MHz, and the input signals are the same as those in the FE simulation. We cut a notch and gradually deepen by a wire electrical discharge machining, then gather data per degree. The notch #1 is 725 mm away from the PZT arrays, and at the long part pf “L” section as #1 in Figure 10. We take the loss ratio of cross-section as a reference value of damage degree

(19) $$D=\frac{S_{d}}{S}\times 100\%,$$

where *S* and $S_{d}$ are the areas of the whole cross-section and damage, respectively. Here we preset four notches (#1-1, #1-2, #1-3 and #1-4) with varying degrees as: 5%, 10%, 15% and 20%, i.e., the length of 2.35 mm, 4.70 mm, 7.05 mm and 9.40 mm. Finally, we can figure out the damage location by the damage echo which can be explicitly identified by the given damage index (DI), the ratio of the damage echo amplitude to the excitation peak amplitude.

In addition, a 3000 mm length L-bar with the same cross-section is implemented as another sample in order to eliminate the uncertainty of the experimental verification. The notch #2 is 750 mm away from the PZT arrays, unlike the previous sample, at the short part of the “L” section as #2 in Figure 10. We still take four measurements with varying damage degrees same as notch #1.

### 4.2. Results and Discussion

**Figure 18:**
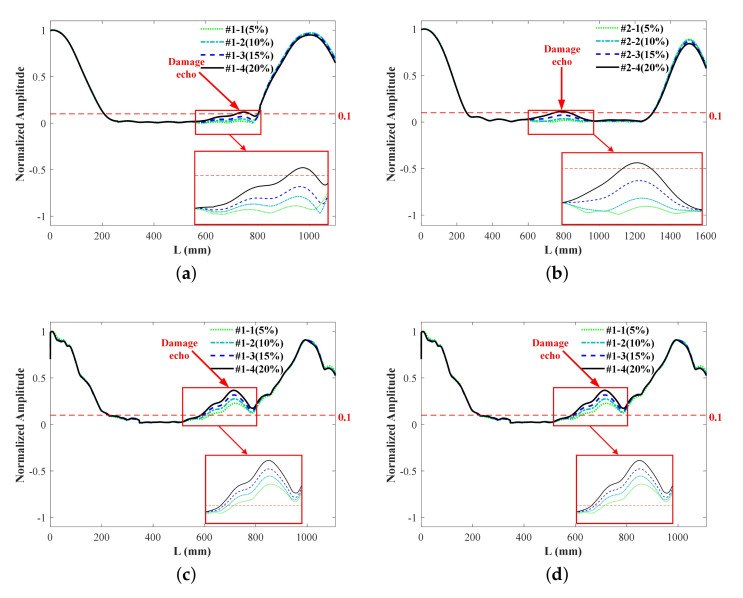
Experimental damage signals of different notches: (**a**) #1 25 kHz, (**b**) #2 25 kHz, (**c**) #1 SoM, (**d**) #2 SoM.

In fact, the group velocity in the experiment is usually a little bit different from the theoretical group velocity due to the difference in material properties [20]. In order to reduce the impact of this difference of group velocity, the Equation (17) is modified as

(20) $$L=\frac{t-T/2}{T_{e}-T/2}\times L_{b},$$

where $L_{b}$ is the length of bar, and $T_{e}$ is the ToF of bar end echo. After the weighted gathering and SoM, damage signals under varied notches of different lengths are shown in Figure 18.

The DI is set to 0.1, then damage echo packets can be observed obviously in the Figure 18c,d. However, damage echo packet beyond DI only can be seen in the case #1-4 and #2-4 in Figure 18a,b, that is, the notch is not diagnosed until its loss ratio of the cross-section has achieved 20%. The SoM of multi-mode ultrasonic guided waves is undeniably conducive to improving the detection sensitivity and reducing the omission. The damage locations are figured out and presented in Table 5, as predicted, the detection accuracy of SoM is higher than low-frequency detection, with localization errors notably narrowed down to 2% and even smaller.

## 5. Conclusions

This paper presents a damage detection method using multi-mode ultrasonic guided waves for L-bars with asymmetric cross-sections. To improve the sensitivity and accuracy of damage detection, the sum of the multiple signals (SoM) method is used to replace the traditional low-frequency detection. Moreover, a mode control strategy is applied to make damage signals of desired modes unacted on multi-mode characteristics, including excitation optimization and weighted gathering based on the semi-analytical finite element (SAFE) and normal mode expansion (NME).

A concise and precise interpretation of the methodology is introduced at the beginning of this paper. Then a proof-of-concept example of L-bar is proposed in detail to validate the proposed damage detection method with finite element simulations and experiments. The eligible frequencies and modes of multi-mode ultrasonic guided waves (25 kHz M4, 80 kHz M9, 100 kHz M11, 155 kHz M13) are selected for the L-bar on the basis of mode characteristic analysis. The mode control using excitation optimization and weighted gathering depended on multielement loading are compared with the only one element loading. As seen from the results, the SoM exhibits higher accuracy and sensitivity to damages than conventional low-frequency detection, which evaluates the effectiveness of the proposed method. The modification of the proposed method for multiple damages and more forms of damage will be studied in our future work.

## Figures and Tables

**Table 1:** Normalized weights.

Transducer Number	Weight at 25 kHz	Weight at 80 kHz	Weight at 100 kHz	Weight at 155 kHz
1	0.9726	0.7181	0.1310	0.6356
2	0.9789	0.9467	0.1255	0.2772
3	0.9818	0.9835	0.1361	0.1111
4	0.9790	0.8389	0.2377	0.3363
5	0.9681	0.8251	0.1958	0.3239
6	0.9842	1	0.1944	0.1294
7	0.9908	0.9467	0.0767	0.2478
8	0.9954	0.1684	0.0692	0.6736
9	0.9966	0.0978	0.2064	0.3287
10	0.9903	0.1645	0.5762	0.2983
11	0.9771	0.0816	0.8560	0.6964
12	0.9565	0.0570	1	0.9959
13	0.9516	0.0879	0.9616	1
14	0.9777	0.1198	0.9131	0.6903
15	0.9944	0.1687	0.5446	0.3084
16	1	0.0530	0.1641	0.3206
17	0.9944	0.1584	0.0004	0.6779
18	0.9791	0.4337	0.0167	0.8351

**Table 2:** Parameter setup in FE simulation.

Parameter	Setup
Analysis type	Dynamic, Implicit
Step	0.1 μs
Element size	0.5 mm (PZT)
	2 mm (plate)
Element type	C3D8E (PZT)
	C3D8 (plate)

**Table 3:** The material properties of PZT.

Density	Young’s Modulus	Electrical Permittivity	Piezoelectric
7650 kg/m${}^{3}$	80 Gpa	D11 = 1.0005$\times 10^{-8}$ Farad/m	−3.09 N/Volt·m
		D22 = 1.0005$\times 10^{-8}$ Farad/m	
		D33 = 8.0926$\times 10^{-9}$ Farad/m	

**Table 4:** FE damage localization results.

Damage	Signal	Location	Absolute Error	Relative Error
#1	25 kHz	473.7 mm	26.3 mm	5.26%
	SoM	498.8 mm	1.2 mm	0.24%
#2	25 kHz	471.3 mm	28.7 mm	5.74%
	SoM	501.9 mm	1.9 mm	0.38%

**Table 5:** Experimental damage localization and error results.

Damge Position / End Position	Damage	25 kHz	SoM
725 mm / 1000 mm	#1-1(D5%)	/	715.0 mm 1.38%
	#1-2(D10%)	/	715.8 mm 1.27%
	#1-3(D15%)	/	717.2 mm 1.08%
	#1-4(D20%)	745.8 mm 2.86%	719.8 mm 0.72%
750 mm / 1500 mm	#2-1(D5%)	/	765.4 mm 2.05%
	#2-2(D10%)	/	754.7 mm 0.63%
	#2-3(D15%)	/	753.5 mm 0.47%
	#2-4(D20%)	787.1 mm 4.95%	752.2 mm 0.29%

## Data Availability

Not applicable.

## Appendix A

### Appendix A.1

This appendix is a extra section that contains details of methods or techniques supplemental to the main text. Here are the key steps of the Matlab code for Section 3.2.

**Algorithm 1:** SAFE and NME.
1: **Input** properties, parameters of structures 2: **Assemble** stiffness and mass matrices, get K${}_{1}$, K${}_{2}$, K${}_{3}$, M 3: **FOR** $k=k_{\min}:▵k:k_{\max}$ 4: **Procedure** SAFE 5: **Solve** $\left[K_{1}+ikK_{2}+k^{2}K_{3}-\omega^{2}M\right]\tilde{U}=0$ 6: **Get** $\omega_{+}$,$U^{j+}$ 7: **Evaluate** $C_{p}$,$C_{g}$ to get dispersion curves 8: **Plot** mode shapes 9: **Plot** dispersion curves 10: **END** FOR 11: **Input** loadings 12: **FOR** $m=k1:1:N_{\mathrm{mode}}$ 13: **Procedure** NME 14: **Calculate** Pm 15: **Calculate** amplitude factor $\langle p_{1}\left(x\right),e^{jk_{m}x}\rangle$ and $\langle p_{2}\left(y,z\right),v_{m\gamma }^{*}\rangle$ 16: **Calculate** amplitude 17: **END** FOR 18: **Bar plot** normalized amplitudes

### Appendix A.2

This appendix is a extra section that contains outputs of every receiving PZT in undamaged FE simulation in Section 3.3.

## References

[1] Mei H., Giurgiutiu V. (2021). High-order wave-Damage interaction coefficients (WDIC) extracted through modal decomposition. Sensors.

[2] Giurgiutiu V. (2005). Tuned lamb wave excitation and detection with piezoelectric wafer active sensors for structural health monitoring. J. Intell. Mater. Syst. Struct..

[3] Su Z., Ye L., Lu Y. (2006). Guided lamb waves for identification of damage in composite structures: A review. J. Sound Vib..

[4] Wang Y., Gao L., Yuan S., Qiu L., Qing X. (2014). An adaptive filter-based temperature compensation technique for structural health monitoring. J. Intell. Mater. Syst. Struct..

[5] Mei H., Haider M., Joseph R., Migot A., Giurgiutiu V. (2019). Recent advances in piezoelectric wafer active sensors for structural health monitoring applications. Sensors.

[6] Gao D., Wu Z., Yang L., Zheng Y. (2016). Guide waves-based multi-damage identification using a local probability-based diagnostic imaging method. Smart Mater. Struct..

[7] Liu K., Wu Z., Jiang Y., Wang Y., Zhou K., Chen Y. (2016). Guided waves based diagnostic imaging of circumferential cracks in small diameter pipe. Ultrasonics.

[8] Mei H., Haider M., James R., Giurgiutiu V. (2020). Pure S0 and SH0 detections of various damage types in aerospace composites. Compos. Part B-Eng..

[9] Qing X., Li W., Wang Y., Sun H. (2019). Piezoelectric transducer based structural health monitoring for aircraft applications. Sensors.

[10] Kamal A.M., Lin B., Giurgiutiu V. (2014). Exact analytical modeling of power and energy for multimode lamb waves excited by piezoelectric wafer active sensors. J. Intell. Mater. Syst. Struct..

[11] Raghavan A., Cesnik C.E. (2005). Finite-dimensional piezoelectric transducer modeling for guided wave based structural health monitoring. Smart Mater. Struct..

[12] Mei H., Giurgiutiu V. (2019). Guided wave excitation and propagation in damped composite plates. Struct. Health Monit..

[13] Ditri J.J., Rose J.L. (1992). Excitation of guided elastic wave modes in hollow cylinders by applied surface tractions. J. Appl. Phys..

[14] Li J., Rose J.L. (2001). Excitation and propagation of non-axisymmetric guided waves in a hollow cylinder. J. Acoust. Soc. Am..

[15] Zhou C., Zhang C., Su Z., Yue X., Xiang J., Liu G. (2017). Health monitoring of rail structures using guided waves and three-dimensional diagnostic imaging. Struct. Contr. Health. Monit..

[16] Fan Z., Lowe M.J.S., Castaings M., Bacon C. (2008). Torsional waves propagation along a waveguide of arbitrary cross section immersed in a perfect fluid. J. Acoust. Soc. Am..

[17] Zuo P., Yu X., Fan Z. (2020). Acoustoelastic guided waves in waveguides with arbitrary prestress. J. Sound Vib..

[18] Li F., Li H., Qiu J., Meng G. (2017). Guided wave propagation in H-beam and probability-based damage localization. Struct. Contr. Health. Monit..

[19] Yu X., Fan Z., Castaings M., Biateau C. (2017). Feature guided wave inspection of bond line defects between a stiffener and a composite plate. NDT E Int..

[20] Zhang J., Wu Z., Yang Z., Gao C., Liu K., Zheng Y., Zhou K. (2020). Multimode guided waves-based structural defect localization longitudinally and cross-sectionally in T-bars. J. Aerosp. Eng..

[21] Ma Y., Yang Z., Zhang J., Liu K., Wu Z., Ma S. (2019). Axial stress monitoring strategy in arbitrary cross-section based on acoustoelastic guided waves using PZT sensors. AIP Adv..

[22] Michaels J.T., Lee S.J., Croxford A.L., Wilcox P.D. (2013). Chirp excitation of ultrasonic guided waves. Ultrasonics.

[23] Giurgiutiu V., Bao J. (2004). Embedded-ultrasonics structural radar for in situ structural health monitoring of thin-wall structures. Struct. Health Monit..

[24] Sternini S., Quattrocchi A., Montanini R., Pau A., Lanza di Scalea F. (2017). A match coefficient approach for damage imaging in structural components by ultrasonic synthetic aperture focus. Procedia Eng..

[25] Shan S., Qiu J., Zhang C., Ji H., Cheng L. (2016). Multi-damage localization on large complex structures through an extended delay-and-sum based method. Struct. Health Monit..

[26] Rose J.L., Lissenden C.J. (2014). Guided wave mode and frequency selection tips. AIP Conf..

[27] Hayashi T., Song W.J., Rose J.L. (2003). Guided wave dispersion curves for a bar with an arbitrary cross-section, a rod and rail example. Ultrasonics.

[28] Hayashi T., Tamayama C., Murase M. (2006). Wave structure analysis of guided waves in a bar with an arbitrary cross-section. Ultrasonics.

[29] Yang Z., Wu Z., Zhang J., Liu K., Jiang Y., Zhou K. (2019). Acoustoelastic guided wave propagation in axial stressed arbitrary cross-section. Smart Mater. Struct..

[30] Ahmad Z.A. (2011). Numerical Simulations of Lamb Waves in Plates Using a Semi-Analytical Finite Element Method. Ph.D. Thesis.

[31] Fritsch F.N., Carlson R.E. (1980). Monotone piecewise cubic interpolation. SIAM J. Numer. Anal..

[32] Rose J. (2014). Ultrasonic Guided Waves in Solid Media: Plates.

[33] Loveday P.W., Long C.S., Ramatlo D.A. (2018). Mode repulsion of ultrasonic guided waves in rails. Ultrasonics.

[34] Zhang J., Xu H., Zhou K., Yang Z., Liu K., Zheng Y., Ma S., Wu Z. (2021). Baseline-free damage diagnostic imaging approach relying on the extraction of converted modes of ultrasonic guided waves. J. Aerosp. Eng..

[35] Zhang J., Wu Z., Yang Z., Liu K., Zhou K., Zheng Y. (2020). Excitation of guided wave modes in arbitrary cross-section structures by applied surface tractions. Smart Mater. Struct..

[36] Auld B.A. (1990). Acoustic Fields and Waves in Solids.

[37] Wu J., Tang Z., Lü F., Yang K. (2018). Ultrasonic guided wave focusing in waveguides with constant irregular cross-sections. Ultrasonics.

[38] Manbachi A., Cobbold R.S.C. (2011). Development and application of piezoelectric materials for ultrasound generation and detection. Ultrasound.

[39] Moser F., Jacobs L.J., Qu J. (1999). Modeling elastic wave propagation in waveguides with the finite element method. NDT E Int..

[40] ABAQUS (2014). Abaqus User Manual, Version 6.14.

[41] Ma S., Wu Z., Wang Y., Liu K. (2015). The reflection of guided waves from simple dents in pipes. Ultrasonics.

